# Successful rechallenge with cetuximab after an infusion related reaction to panitumumab in a patient with locally advanced rectal cancer

**DOI:** 10.1007/s13691-020-00455-x

**Published:** 2020-10-30

**Authors:** Hideyuki Yokokawa, Teppei Kono, Hiroaki Shidei, Kunihiro Oyama, Yoshitomo Ito, Rie Imaizumi, Yutaka Miyano, Shunichi Shiozawa, Kazuhiko Yoshimatsu

**Affiliations:** 1 Department of Surgery, Saitama-ken Saiseikai Kurihashi Hospital, 714-6 Koemon, Kuki, Saitama 349-1105 Japan; 2 Department of Surgery, Tokyo Women’s Medical University, Medical Center East, 2-1-10 Nishiogu, Arakawa, Tokyo 116-8567 Japan

**Keywords:** Infusion related reaction, Panitumumab, Cetuximab

## Abstract

Incidence of infusion related reaction (IR) is more common with cetuximab (Cmab) than with panitumumab (Pmab). Although little is known about rechallenge IR with monoclonal antibodies, we experienced a successful rechallenge to Cmab after IR to Pmab. A 67-year-old female patient was scheduled for chemotherapy with mFOLFOX6 plus Pmab against unresectable advanced rectal cancer in the hope of tumor shrinkage. On the first administration of Pmab, she complained of dyspnea with shortness of breath and wheezing, even after premedication with steroids and antihistamines. Her reaction was judged as Grade 2 IR to Pmab. For the next course, we tried Cmab. No IRs were observed. Since then, she has undergone seven further courses of treatment, followed by surgical resection. The patient benefited from administration of Cmab after experiencing IR to Pmab, suggesting this treatment to be an option for patients of this type who experience IR to Pmab.

## Introduction

Cetuximab (Cmab) is a chimeric antibody that binds to epidermal growth factor receptor (EGFR) with murine fraction variable regions [1], whereas panitumumab (Pmab) is the first complete human monoclonal antibody similar to Cmab [2]. They both inhibit the proliferation and differentiation of EGFR-expressing normal and neoplastic cells and cause apoptosis. The incidence of documented infusion related reaction (IR) is more common with Cmab (all grades (G) 15—21%, G ¾ 2—5%) than with Pmab (all G 4%, G ¾ 1%) [3–9]. Although anecdotal reports suggest successful rechallenge with Pmab following IR to Cmab [10], there are few known cases that show the opposite pattern [11].

We herein present a case of locally advanced rectal cancer that was successfully rechallenged with Cmab after IR to Pmab, followed by surgical resection.

## Case report

**Fig. 1:**
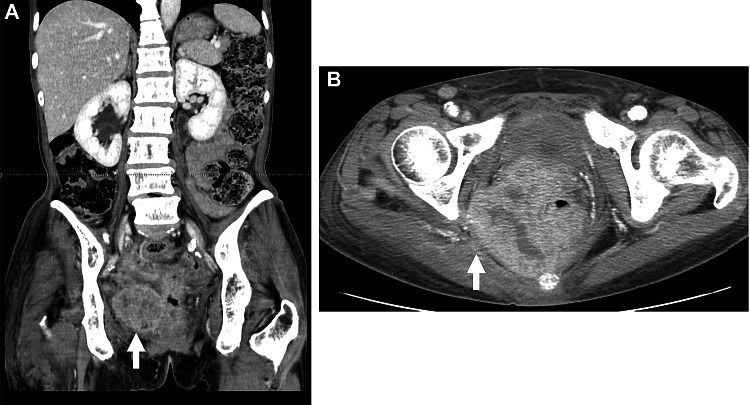
Abdomino-pelvic CT scan on admission. The tumor originating from the rectum had grown into the extra-rectal wall (white arrow: **a**) and was suspected of having invaded the gluteus maximus (white arrow: **b**)

**Fig. 2:**
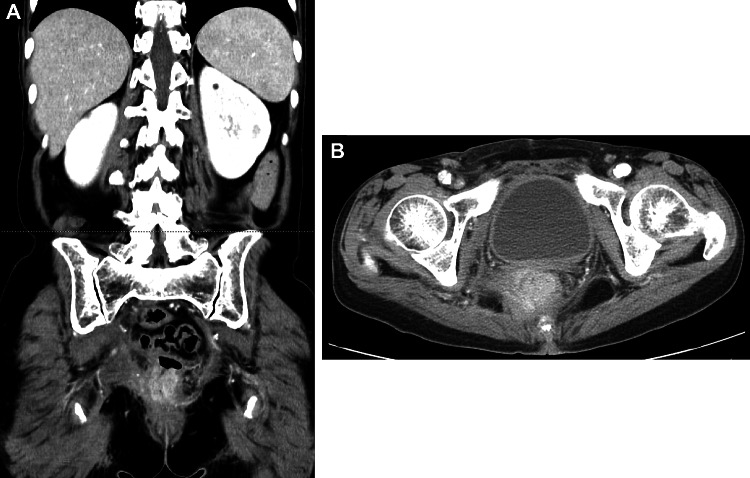
Abdomino-pelvic CT scan after chemotherapy. A marked shrinkage of rectal tumor was demonstrated

A 67-year-old female patient was referred to our hospital with tenesmus and frequent bowel movements. Physical and hematochemical examinations revealed loss of body weight, malnutrition and inflammatory changes. Computed tomography (CT) scan revealed a rectal cancer that had developed to the external wall of the rectum and invaded the surrounding tissue (Fig. 1). On admission, induction of chemotherapy was conducted after stoma creation due to the unresectable nature of the tumor. Due to its identification as a wild-type tumor incorporating rat sarcoma viral oncogene homolog (Ras)/v-raf murine sarcoma viral oncogene homolog B (BRAF) genes, a combination of oxaliplatin, 5-fluorouracil and leucovorin (mFOLFOX6) plus Pmab was selected in the hope of causing rapid tumor shrinkage. As pretreatment before initiation of chemotherapy, 1.65 mg of dexamethasone sodium phosphate and 5 mg of chlorpheniramine maleate were injected to prevent adverse events including allergy. Then 260 mg of Pmab was administered intravenously by drip infusion. The patient then complained of dyspnea with shortness of breath and wheezing. Her oxygen saturation decreased to 88%. Chemotherapy was immediately halted and oxygen, plus 250 mg of aminophylline hydrate and 125 mg of methylprednisolone sodium succinate, were given intravenously. As a result of this treatment, her symptoms gradually resolved within a few hours. Due to her successful recovery from IR, mFOLFOX6 without Pmab was administered the following week. No apparent symptoms were observed after this treatment. Diagnosing that the patient had suffered severe IR to Pmab, concomitant use of Cmab was attempted in the next course while monitoring vital signs. Three hundred and twenty mg of Cmab was slowly infused (2.7 mg/minute) after premedication with 1.65 mg of dexamethasone sodium phosphate, 20 mg of famotidine and 50 mg of Restamine calcium. No abnormal vital signs or IR symptoms were detected during administration. Other drugs were also administered without the appearance of any symptoms. Treatment was continued for seven courses due to no toxicities that might suggest the need for dose reduction or postponement, even though the patient experienced toxicities that included G2 dermatitis, G2 peripheral neuropathy, G2 dysgeusia, G2 thrombocytopenia, G1 anemia and G1 neutropenia. Marked tumor shrinkage (Fig. 2) allowed abdomino-perineal resection to be performed. Pathologically, the rectal wall was highly degenerated and showed fibrotic changes. However, live cancer cells remained. Since these were found close to the surgical margin, she was diagnosed with pT4b (pelvic tissue), pN0, pStage II, pRM1, curB.

## Discussion

Monoclonal antibody treatments that act on EGFR, including Cmab and Pmab, are recommended for Ras wild-type-metastatic colorectal cancer as candidates for first-line to third-line therapy [12–14]. Although cutaneous side effects are common, due to inhibition of EGFR expression in normal organs, certain prophylactic measures are commonly used, such as the use of moisturizing ointments or steroids, and oral intake of minocycline hydrochloride [15].

IR is a less common adverse event caused by antibodies to EGFR [2]. The fact that 90% of IRs occur during the first infusion despite antihistamic premedication suggests that these reactions occur without any IgE-mediated reaction [16]. It is possible that IRs to monoclonal antibodies are a reaction to human antichimeric antibodies or anti-human antibodies. In general, IR is more common with Cmab than Pmab. Possible rechallenge with Cmab may be due to differences in the reacting antibodies, even though no correlation between IR and these antibodies has been demonstrated. Another hypothesis for the mechanism that induces IR is that it is associated with the role of complement activation and the release of cytokines [17].

Our study has several limitations. First, mFOLFOX6 alone had a substantial therapeutic effect, and therefore, the benefit of the addition of Cmab were unclear. But previous study showed the superiority in the response rate of advanced colorectal cancer treated with FOLOX plus anti-EGFR antibody compared to FOLFOX [18, 19]. Therefore, in this study, mFOLFOX plus Cmab potentially contributed to shrink tumor. Second, in patients with resectable colorectal liver metastasis, it is currently questionable whether resection of metastatic lesions after shrinking the tumor using Cmab plus mFOLFOX6 [20, 21]. But this is a report about locally advanced rectal cancer without any distant metastases. Resection of the shrinked primary lesion after chemotherapy containing anti-EGFR antibody might have improved her prognosis.

To our knowledge, this report is the second documentation of a case of successful rechallenge with Cmab after IR to Pmab. Since our case showed tumor shrinkage with concomitant use of Cmab and mFOLFOX6, she was able to undergo radical resection. Although IR is less frequent with Pmab, patients with severe IR to Pmab can be rechallenged with Cmab. However, further studies are needed to elucidate the pathogenesis and mechanisms of antibody-mediated IR and to gauge the safety of re-challenging with the same or different antibodies.
